# Positive postpartum well‐being: What works for women

**DOI:** 10.1111/hex.13605

**Published:** 2022-11-02

**Authors:** Susan Hannon, Elizabeth Newnham, Kathleen Hannon, Francesca Wuytack, Louise Johnson, Ellen McEvoy, Déirdre Daly

**Affiliations:** ^1^ School of Nursing and Midwifery, Trinity College Dublin The University of Dublin Dublin Ireland; ^2^ School of Nursing and Midwifery, Trinity Centre for Maternity Care Research (TCMCR) Trinity College Dublin Dublin Ireland; ^3^ School of Nursing and Midwifery University of Newcastle Callaghan New South Wales Australia; ^4^ ESTER Team—Epidemiology in Occupational Health and Ergonomics, Department of Medicine, Research Institute for Environmental and Occupational Health (IRSET—Inserm UMR 1085) University of Angers Angers France; ^5^ Study Participant, c/o Susan Hannon, School of Nursing and Midwifery Trinity College Dublin Dublin Ireland

**Keywords:** maternal health, postnatal, postpartum period, qualitative research, salutogenesis, well‐being

## Abstract

**Background:**

Women's experiences of pregnancy, birth and motherhood extend beyond healthcare provision and the immediate postpartum. Women's social, cultural and political environments shape the positive or negative effects of their experiences through this transition. However, there is limited research concerning the factors that women identify as being protective or promotive of maternal well‐being in the perinatal period and motherhood transition.

**Objective:**

To explore women's views on the factors within healthcare, social, cultural, organizational, environmental and political domains that do or can work well in creating positive perinatal experiences.

**Design, Setting and Participants:**

A qualitative descriptive study with embedded public and participant involvement (PPI). Participants were 24 women who were maternity care service users giving birth in Ireland.

**Results:**

Three themes were developed. The first theme, ‘tone of care’, related to women's interactions with and attitudes of healthcare professionals in setting the tone for the care they experienced. The second theme, ‘postpartum presence and support’, concerned the professional postpartum supports and services that women found beneficial in the motherhood transition. The final theme, ‘flexibility for new families’ addresses social and organizational issues around parents returning to paid employment.

**Discussion and Conclusion:**

Women suggested multiple avenues for promoting positive perinatal experiences for women giving birth in Ireland, which may be implemented at healthcare and policy levels. Women identified that maternal health education focuses on supporting informed decision‐making processes as a positive and worry‐alleviating resource. Additionally, women valued being met by healthcare professionals who regard women as the decision makers in their care experience. Exchanges in which healthcare professionals validate and encourage women in their mothering role and actively involve their partners as caregivers left lasting positive impressions. Extended and professional postpartum support was a common issue, and phone lines or drop‐in clinics were suggested as invaluable and affirming assets where women could access personalized support with healthcare professionals who had the knowledge and skills to genuinely approach women's concerns. Social and organizational considerations involved supporting parents to balance their responsibilities as new or growing families in the return to work.

**Public or Patient Contribution:**

Maternity care service users were involved in the interviews and manuscript preparation.

## 1 INTRODUCTION

A positive experience of pregnancy, childbirth and the transition through motherhood is a highly desirable outcome for all women. It is an indicator of effective maternity care and services and necessary for the well‐being of the mother, child and family unit, with wide‐reaching social ramifications. There has been a notable widening of attention within health research, policy and practice over the past two decades beyond the predominant focus on mortality and morbidity. Scholars increasingly acknowledge that a complete understanding of health must involve a holistic consideration, through the inclusion of positive health and well‐being, not simply denoting the absence of illness. This consideration is reflected in the World Health Organization's (WHO) antenatal, intrapartum and postnatal care recommendations.^1^, ^2^, ^3^ A consolidation of the WHO's definitions of ‘positive care experiences’ within these documents indicates that positive care during these periods involves (i) the fulfilment or surpassing of women's personal and sociocultural beliefs and expectations, (ii) a healthy pregnancy, baby and mother, (iii) continuity of emotional and practical support from birth partners, (iv) consistent reassurance and support from competent staff and (v) a resourced and flexible health system.^1^, ^2^, ^3^

Postnatal care and services have been described as the neglected aspect of maternity care throughout the world for decades. Criticisms include level and scope of service provision, duration of care and quality of care.^4^, ^5^, ^6^, ^7^, ^8^, ^9^, ^10^, ^11^ However, some studies have identified positive aspects of care, including active practical guidance with infant care and feeding,^12^ assistance‐focused, nonjudgemental at‐home postpartum visits from a healthcare worker^13^ and home‐based postpartum care.^7^ Additionally, positive postnatal care was found to enable women to adapt to their new identity, become confident in mothering and know and understand the physical and emotional changes that accompany childbirth and motherhood.^14^

Evidently, perinatal care and quality services play pivotal roles in supporting women's perinatal health; however, women's experiences of pregnancy, birth and motherhood extend beyond healthcare provision. Women's social, cultural and political environments shape the positive or negative effects of their experiences through this transition.^15^, ^16^, ^17^ Strong social support is an often‐cited buffer against negative outcomes in any context, and it appears that in relation to the perinatal period, social support is the predominant area of exploration for promotive and protective factors for mental well‐being.^18^ There is, however, limited research concerning the factors that women identify as being protective or promotive of maternal well‐being. Specifically, the factors that are present within women's interpersonal, social, ecological and political contexts and the healthcare systems available to them that (i) soothe or allay women's fears, worries, concerns and anxieties in the transition to motherhood and (ii) help women to adapt into this transition, support their confidence and well‐being and promote a positive experience of the perinatal period.^19^, ^20^ The current study aimed to develop a deeper understanding of how the perinatal period can be experienced positively, by centring women's voices and lived experiences concerning the impact of healthcare, social, cultural, organizational, environmental and political factors on their well‐being during this transition. In short, the study sought to explore women's views on what factors worked well, and/or what could work well, in creating a positive perinatal experience.

## 2 METHODS

### 2.1 Design

A qualitative study with embedded public and participant involvement (PPI) explored the maternal health‐related issues that matter most to women in the transition to motherhood, based on their experiences of maternity care and services.^21^ The study was a substudy of the MAMMI (Maternal health And Maternal Morbidity in Ireland) study, a longitudinal study investigating the health and health problems of 3047 first‐time mothers in Ireland.

The current study is a secondary analysis of data from women who participated in the PPI qualitative substudy.^21^ The substudy identified a central concept of the ‘invisible woman’, which encapsulated issues arising within the current Irish maternity care system that left women feeling unheard, unseen and uncared for. The purpose of the current study was to tease out what women said worked well and/or what could have worked well when their experiences were less than positive. To achieve this, we conducted a secondary analysis of the data from the original PPI data to identify what factors support/could support women, within and beyond the health and maternity care systems, and foster positive experiences of the transition to motherhood. Findings are presented with this aim in mind; indications of less than optimal care or experiences are contrasted with solution‐based descriptions of what women said did or could have succeeded in making their experience a positive one. This approach was derived from the way that women often spoke about their experiences, which were often accompanied by a description of care, services or resources that they wished they received/had access to.

### 2.2 Ethics

The research ethics application was drafted by the researchers and reviewed by five women in the PPI study. Ethical approval was granted by the Faculty of Health Sciences' Research Ethics Committee of Trinity College Dublin. All participants completed written informed consent. Participants' identities have been anonymized for publication and all names are pseudonyms.

### 2.3 Recruitment and participants

Following ethical approval, a letter of invitation was emailed to all MAMMI study participants who consented to receive research information and invitations. Women interested in taking part contacted the researchers directly by email or text; they then received a study information pack. Recruitment took place in June 2018.

Interviews with 24 women were conducted between June and September 2018 by one interviewer (Table 1). Details on recruitment and data collection were described previously.^21^

**Table 1:** Participants' characteristics

Age	*n*	Marital status	*n*	Ethnicity	*n*	Education^a^	*n*	Employment^a^	*n*
30–35	9	Married	18	White Irish	20	Postsecondary level	4	Full‐time paid employment	21
36–40	10	Living with a partner	4	White European	2	Primary degree or equivalent	11	Part‐time paid employment	2
41–45	5	Engaged	2	Irish/Caribbean	1	Postgraduate degree or equivalent	8	Student	1
				Undisclosed	1	PhD	1		

^a^ At the time of first pregnancy.

### 2.4 Data analysis

Data were reflexively thematically analysed using Braun and Clarke's six‐step framework^22^: familiarization; transcribing, reading and rereading the data; generating initial codes; searching for themes; reviewing themes; defining and naming themes and writing the report. Data were managed using Microsoft Excel. Illustrative quotes are presented using pseudonyms.

### 2.5 Rigor

This study is underpinned by a critical realist ontology and a constructionist epistemology and takes a participatory/advocacy approach. It is part of a PPI initiative and a wider study examining Irish women's experiences of motherhood. The researchers are maternal health researchers (midwives, psychologists, sociologists and chiropractors), advocates of women's health and agency, who co‐conceived/co‐designed the study with women in the PPI. This approach influenced the way that data were analysed, and themes were produced. This reflexivity and transparency aid in qualitative research rigour and we present raw data to demonstrate each theme. Co‐authorship with one to two self‐nominating women from the PPI study was planned from the outset.

For the current study, three researchers (S. H., E. N. and D. D.) refamiliarized themselves with the complete interview data set and then analysed eight transcripts each in which they identified new codes related to the secondary research focus. We met to discuss our initial codes and findings and to ensure that there was no overlap with the findings of the previous study. Following the agreement of codes and themes, the final themes and a selection of anonymized illustrative quotations were returned to participants for confirmation and comments. Participants were asked if they recognized their views or the views of others that they may have heard of, even if they did not hold or agree with those views themselves. Four participants responded, and all stated that the findings and themes were very or fairly true of their own views and that they recognized the views of others even if they did not hold those views themselves. Two PPI members (L. J. and E. M.) who were also participants of the original study were involved in refining the final themes and manuscript preparation.

## 3 FINDINGS

Participants' characteristics are presented in Table 1.

### 3.1 Theme 1: Tone of care

This theme describes how aspects of care during the antenatal and early postpartum period may lend a positive or negative ‘tone’ to a woman's perinatal experience. Here, the ‘tone of care’ encompasses the responses, reactions, attitudes and sentiments women perceived towards them from health care professionals, health services and policies for care. The dynamic of exchanges between a HCP and a woman has the potential to inspire feelings of safety and respect within the healthcare services, while also setting a ‘tone’ of what women can hope to expect from care. This theme contains three subthemes: (a) *Respecting women as decision‐makers*; (b) *Family‐focused care*; (c) *Attentive presence of a healthcare professional*.

#### 3.1.1 Respecting women as decision‐makers

It was important for women to feel that *they* were making decisions about their care and that those decisions were directed by an informed understanding of their options. Women spoke about the value of having the HCP recognize their authority in decision‐making through information sharing and honouring women's final decision‐making power. This is exemplified below in one woman's comparison between her first and second pregnancy and her experience in choosing a privately practising midwife for her second pregnancy because she felt she had an unnecessary intervention in her first.

A big issue for me in my first pregnancy was consent. I was diagnosed, I believe, incorrectly with gestational diabetes and then it all went downhill from there … I was forced onto insulin. I was forced into an induction that didn't go very well at all. And I was treated like, basically like a cow in a shed…. In my second pregnancy … I hired my midwife … oh god it's marvellous. It's five thousand euros and it is worth every single cent. You get all your antenatal visits in your house, at your own time. Everything is explained to you. All your options are given to you. Nobody touches you without asking you, you just feel, you feel you're in control of your pregnancy. (Clíodhna)

This sense of control was experienced even though the woman experienced medical interventions during both births—induction of labour with her first and a caesarean section with her second—because of the way that the decision was left to her, facilitated by information sharing that included rich discussion of all courses of action.

And I was able to decide … between us, we were able to sit down and have a chat about it, in our own time. And I felt like, right ‘Now I'm going into having a section, because I know that's the safest thing to do’. (Clíodhna)

Without this sense of being informed and empowered, women felt removed from the decision‐making process.

I was, again, perhaps naive and perhaps vulnerable and uncertain and presuming that this person who spoke to me was acting in my best interest and maybe she was, but … I would have liked someone to have maybe said ‘well, take half an hour and think about it, here's what's involved if you do this and here's why we're recommending it, but these are your options’—or ‘you don't have any options and this is the only course of action’. (Camilla)

‘I only know after having a second baby, em I had to have a section again, that you actually do have options of how they operate and apparently I was a lot more vocal because I had spoken with more people about it but they don't provide that information there, they just tell you “this is what's happening and deal with it,” basically’. ‘Even though I'd probably still make that decision today, you're not actually … nothing is explained to you about what will happen afterwards, or what the implications are afterwards’. (Caitlynn)

Some women compared their hospital‐based antenatal education with an external non hospital‐based antenatal education course. For some women, the external course was helpful in reminding them that they should have the final say over decisions about their bodies during labour and birth. Women mentioned that the information given from the hospital, while practical in terms of baby care information, did not provide the information they wanted about the various common scenarios that may arise. The external course was delivered outside the standard hospital antenatal education classes and stood out as an educational model that encouraged women to ask questions about their care and options.

(The hospital class) was a lot of common sense stuff and what to bring to the hospital and all that sort of thing. Whereas the other one … was more empowering, about understanding the whole medical side of things, so that you were more in charge of your own labour and delivery. Rather than just feeling like you were … I was going to say, prey for the consultants but you know? That you were secondary, you were just, everybody else was in charge and you were just … (laugh). (Olivia)

The external course also clearly recognized the role, importance and involvement of the partner. As one participant identified, it empowered her partner to take an active role in the labour process.

The … course; it empowered me. But definitely, my husband, like he was very active in the labour. And whereas, I think he would've maybe just kind of, wouldn't have known his place. And what his role was really apart from just being there you know? So I definitely think it was beneficial in that sense. (Eimear)

#### 3.1.2 Family‐focused care

Women spoke about how (i) processes that included their partners and (ii) interactions that demonstrated that HCPs equally regarded their partners as a caregiver to their child improved their experiences. Partners in this sample were all men/fathers but this finding would be applicable to other family situations.

He was involved in it all. He was treated very well by the staff … They also like had him dress the baby, well he didn't know how, but they did show him how to dress the baby. And then when we went back up to the ward, they also showed him, told him to get all my stuff out on the table and ready … And my partner would be very nervous around new babies. (Karolina)

Recognition of a partner as a parent and caregiver meant that women felt less burdened by dated perceptions and expectations around gender roles and childcare responsibilities.

I still felt that it was very much focused on me and part of that brings a little bit of a pressure in that, it's all on me when they're looking to me for all of the decisions and it's not that I didn't want it but it certainly feels like it's my responsibility distinct from a shared responsibility with my husband. (Isabel)

The attitude that I encountered while I was there. It was not even just the lack of support. It was like women should be on top of it. That is their job. And if the man wants to get involved clearly, there's something wrong with her. Because she's not capable. And that's just not a good attitude. (Morgan)

#### 3.1.3 Attentive presence of an HCP

Having the focused presence of an HCP that shared their expertise and offered guidance to a new mother was often key to women feeling supported and could help boost women's confidence in their new role.

When it comes to the first‐time mums, you can't just say your instincts are going to kick in, you know, and ‘trust your gut’ and all of these kinds of things. I think someone needs to sit with a mum for an hour, maybe 2 hours and just literally go ‘ok we're going to change this first nappy, I'm not going to do it for you, but I'm going to be here and I'm going to be supporting you if you need me’. (Alex)

Women were sympathetic to issues of workload and staffing, particularly within the postnatal ward; however, even brief interactions were described as valuable, as feedback and guidance provided validation and reassurance to women that they were doing the right thing. The quote below demonstrates how one woman felt when no HCP stopped to provid support with infant feeding and the incongruity with the hospital's policy/advertisements.

(It's) time for them to be like, okay how do we support women? And how do we set them up? And how do we give them the confidence that they might need to move forward, without judging them? But there was literally like … it just made me laugh, there are posters all over the place around breastfeeding. And not one person, not one person offered to help. (Morgan)

For one woman, even a few minutes of a midwife's time was deeply appreciated and the quote below illustrates that this focused attention does not need to take hours of time.

One midwife, and she was the only one that actually stopped and gave me a couple of minutes of their time. I was struggling, I was trying to, to feed [baby] sitting up in the bed and she, like, what she did was nothing really, but she came over and she stood beside me and she said; you can lie down and feed him lying, and I didn't know that … what I needed was … somebody who was willing, and she was literally rushing … past me, and she saw me really struggling and she was kind enough to stop for the two minutes that she did, so that's what I would have needed, was someone just to sort of say ‘are you doing okay?’. (Grace)

### 3.2 Theme 2: Postpartum presence and support

Women described the wealth of professional support during pregnancy to its near or total absence postpartum as jarring, perhaps because it clearly announced that this interest was for the sake of the baby which, once the baby was born, stopped suddenly.

Did they just see me as a vessel? Like was I just there … the only reason they're actually minding me was to mind the baby? And I get that, I 100% get the importance of that, I really do. But like. It's like the second the baby has left your body, literally. They have zero interest in you as a person, zero interest in how you're doing. (Morgan)

Although maternal healthcare may conclude at 6 weeks postpartum, this is an arbitrary cut‐off for women whose navigation of life with a newborn continues after formal support have ceased. The sense that women really wanted and benefited from support and reassurance was developed into the second theme and two subthemes: (a) *Someone to ask, someone who asks, someone who listens* and (b) *Somewhere to go.*

#### 3.2.1 Someone to ask, someone who asks, someone who listens

Women frequently mentioned the need for a professional point of contact when at home postpartum. They wanted to be able to ask questions and raise their concerns with an HCP who was flexible, accessible and approachable. For some women, a midwife or public health nurse readily established themselves as this contact. This connection proved beneficial for women who received it and potentially diverted women from accessing more acute services when they did not need to.

‘The public health nurse we had here, and the midwife was amazing … actually my public health nurse, I need to actually write in somewhere about her, because she is so amazing … I couldn't fault her like. Straight away she said, “I think it's more than mucus, I think it's reflux. You need to go to your GP” and stuff like that. And when I'd go to the GP, I'd come back to her and liaise with her. And she'd say “I think it's more, but listen, you know” and I felt like she was the only one that had any fight for me, for my son, like for any issues we had … and I continued to … use her until his last check. I'd ring her like on a regular occurrence and ask her advice’. ‘I found it great because I think as well as that, like I probably would've gone to A&E with my son a lot more. If I hadn't have had her to tell me what was normal and not normal’. (Karolina)

For one participant, this was made clear in its absence:

What I wanted to ask was … is it almost guaranteed to (happen) in the future if I do have a subsequent pregnancy? Maybe, how long should I leave it? I really wanted to ask all those kinds of questions but at that point I was questioning myself and my ability to parent. (Alex)

Others suggested a point of contact as a hypothetical to address some of the issues they found after having their baby.

A professional ‘on the other side of that phone (who) is going to put you in contact with who you need to be in contact with or the relevant service and that there's an understanding that you're not left on hold for an hour (…) that there's some kind of a level of support around all of the elements of care, whether that's just ringing a telephone number to try and get help, that there's an understanding there around the experiences of being a new mum’. (Isabel)

Along with having someone to call, women valued having someone who asked about their health. Limited and baby‐focused postpartum care meant that meaningful follow‐up care focused on the woman was often absent from experiences, as is evident from one participant who simply wanted to be asked how she was doing.

There was nothing, nothing, along those lines at all. And I just find that really a gap as far as the care. Just a genuine, ‘how are you? How are you feeling today? Can we help? Is there anything we can do?’ and not focusing so much on the baby, and again baby is hugely important. But like the mother is too. (Morgan)

On the other hand, women who experienced regular enquiries specifically about *their* health and well‐being from the HCP felt that their health was a priority beyond their role as a mother and provided a sense of support.

She did focus on both of us, she was constantly asking; how are you? And she was saying, you seem to be coping well but how are you feeling? And she wouldn't just assume that you're fine, she'd keep checking with you. She'd let you know when she's calling again, she'd set the date with you so you'd know when to expect her again. It just, felt that there was someone around. (Caitlynn)

Women found it reassuring to have genuine enquiry followed by genuine listening. Being taken seriously by the HCP, being listened to and feeling heard was fundamental to women feeling supported in their transformation into confident mothers.

Luckily, I went to my GP for the six‐week check‐up. And I just said to her, look I don't feel right, I don't feel the same as I did after the first baby. And she was like, ‘Well you know your own body’. And she was fantastic. (Silvia)

Women described the heartening influence of positive validation of their own concerns or opinions from the HCP. Supportive words from HCPs alleviated women's anxieties about the well‐being of their children and their abilities as a mother.

Just to have somebody to tell you that you're doing okay … and that was, I suppose, very important for me, that somebody that knew about babies was kind of (saying) ‘your baby is okay’. (Camilla)

My GP, she was great I have to say. Because I was saying, oh I feel like I'm neglecting them and I'm not doing this and I'm not doing that. And I came in the following week with the kids. And she took one look and she said, ‘so these are the happy, flying, chubby, healthy, neglected children are they?’. (Clíodhna)

#### 3.2.2 Somewhere to go

Face‐to‐face support was a benefit for many women, and whilst some women were able to attend postpartum breastfeeding clinics in the community and avail of support/advice, albeit ad hoc, similar services were not available to women who were not breastfeeding. Services such as these could be confidence‐inspiring for all women.

I started going down to the clinic after a few months and it was just totally different, you felt she was on the end, like she gave me her mobile number and she was there at the end of the phone and she'd give you loads of leaflets or information and she always checked how you are and it was totally different. (Caitlynn)

Women suggested one‐stop postnatal centres as a potential solution:

I know in [hometown], we have … Where we go to see the public health nurse. It's the same as the way there's a mental health clinic setup. In the centres there should be like maybe a postpartum clinic setup. With a gynaecologist that knows what they're doing. Also with a paediatrician, so you're actually seeing specialists in their fields. That it's not going to take long, I'd say half an hour per person … If you have at least one chance of that in the first year of your child's life, I think it would be fantastic. (Karolina)

### 3.3 Theme 3: Flexibility for new families

Women described the real‐life challenges of deciding to return/not return to paid employment, after maternity leave. For many, prohibitive childcare costs or limited service provision left them without options. The key supportive factors identified by women included having a degree of flexibility in returning to work and flexibility for their partners.

#### 3.3.1 Affordable, close‐to‐home childcare

Many women wanted to return to work but the high costs of childcare in Ireland made returning unrealistic and unaffordable. Balancing the consequences of returning to work with the costs of childcare presented only lose–lose outcomes for some women.

My wages wouldn't cover childcare costs at all … Like it wouldn't even come close to it, I'd be literally working for ten euros a day. For somebody else to raise my child and I just couldn't do that. (Karolina)

There wasn't an option to work part‐time in my place either, that's what I had wanted at that stage and it's just a struggle and it's a constant juggle of childcare and I'm always thinking how can I do this or that, or shorten it down. (Brigid)

Few women relayed positive experiences in securing childcare, and those who did described finding a childcare solution almost by luck.

It's not easy to find what you want. And it's very expensive and it's stressful … And we were very lucky because we got a childminder, who had minded my friend's children, very close to the school where I teach. And she's still our childminder now, so we're really lucky that she's been with us for that long. (Leann)

#### 3.3.2 Flexibility for parents returning to work

Greater job flexibility for women and their partners made the return to employment more manageable. At a practical level, a meaningful length of paid paternity leave and flexibility within a partner's occupation benefited mothers. Paternity leave enables partners to engage in and adjust to their new parental role, which supports the new mother.

I was in that very lucky position (…); he's not restricted to nine to five working hours, so he was able to work around my return to work and he was able to work around looking after [child's name]. So we didn't need to pay for childcare for the two days that I was working part time. We didn't have any family to rely on for childcare, but we didn't need it, we managed between ourselves. (Camilla)

Women viewed paternity leave as a societal recognition that a partner (in this study, all partners were male) is as equal a parent as the mother.

The woman gets six months off, like why can't the husband get half of that time off? I know they've changed the law in that. Again, it's just a cultural thing like (…). Like, they're just not, they're secondary. (Sarah)

Women also acknowledged certain perceptions, barriers and career consequences to partners availing of paternity leave.

He would love to have the opportunity to be able to take some time, to actually look after the baby. But you know it's just, it's nearly career suicide for a man if it's even suggested. That shouldn't be the case. It should be just, if a male wants to do it, it should be encouraged and supported. (Eimear)

Several women decided to return to work on a part‐time basis or with decreased hours and described this decision as in the best interest of their family and their own well‐being.

My boss was quite flexible … And I had said to her like, I want a day with my family … [and] … I feel that Sunday is the only day me, my partner and my son could stay together…. So we came to a thing that I'd work every third Sunday. So we did come to an agreement. (Karolina)

For some, the inability to work reduced hours due to financial barriers or inflexibility with work caused deep distress. Providing for their family's needs inevitability affected the time available to women to spend with their family and engage in their parenting role. As one woman described her full‐time return to employment following the birth of her second child:

I'd two children then, it was very, very, very hard. Like really, I think awful actually. It is like; it's depressing like you know what I mean. You're there, you talk to your children and they don't get to see you and you're working full time. And it's absolutely terrible. (Sarah)

Women who were able to avail of a more flexible return to work described multiple benefits for their family; (i) it provided a continued stream of income, (ii) and starting childcare offered their child an avenue for learning social skills and building social connections. Additionally, a flexible return to work supported women's well‐being in that it enabled women to engage more fully in the mothering role by providing a space and sense of identity beyond the mothering role.

My girls love it, like they (friends at crèche) love the girls, and I love my job, and I know I'm a much better mum by doing the way I do it and getting some time for myself. (Morgan)

I did appreciate that time for myself and just … I think the little bit of separation was probably healthy, but it would have been nicer to be able to combine it in a way that felt conducive to family life. (Isabel)

Some women suggested a social/organizational policy that would support women to adjust to managing multiple roles;

Even if there was kind of a transition programme in or like just say for women returning back. That maybe for the first month that they would go back three days a week. And then maybe the second month, it'd be up to four days. And then gradually build themselves back up to five days, if they're going back full time. Because I think it's a huge jump going from having all your time with your little one. (Eimear)

If people could come back and do a 4 day week just for the first 6 weeks even, just to set yourself up like that. So that you can take a day off ad hoc if the child is sick or you're exhausted. (Olivia)

## 4 DISCUSSION

Our findings show that women's descriptions of what works and what could work in creating a positive perinatal experience spanned the pregnancy and postpartum continuum and extended beyond it; to returning, or not, to paid work. The issues evident within the data mainly pertained to healthcare, social, organizational and policy factors, which impact women's experiences.

The findings show that psychological and relational aspects of care, how care is delivered and how information is communicated, can create a positive ‘tone’ to women's overall experience. At the very core of positive maternity care was the experience of women being placed at the centre of their care, in terms of the informational/educational services they received and the attitudes they encountered from HCPs. Having situationally relevant information so that women could make decisions from a position where they felt that their consent was informed was vital to positive experiences. The differences between women's experiences of attentive care during pregnancy and birth and their experiences of ‘baby‐focused’ postpartum care were stark and left many women feeling unsupported. Others have shown that women's maternity care is unbalanced with an antenatal focus and, according to women, improvements in care could be achieved simply by providing time for women postnatally.^23^ For one woman in our study, even a few minutes from a midwife was worthy of mention and made a positive difference.

When women had positive interactions, they felt reassured, validated, heard and taken seriously by an HCP and felt regarded as the expert on their bodies and lived experiences. Similar to other research,^24^ women valued and felt validated in these constructive interactions and, in many instances, this gave them confidence in their ability as a mother. The active presence and practical support of HCPs were pivotal to women's positive experiences, particularly in the early days of motherhood. Women wanted time from HCPs to learn from them and be guided by them. When HCPs were unavailable, women were left feeling uncertain, unconfident and self‐doubting. Time and organizational barriers to providing high‐quality services to women in their care are an issue close to the heart of many midwives and are increasingly documented as a source of moral distress.^25^ Women understood the demands placed on HCPs, however, descriptions of interactions, which set a positive tone in their experiences, often involved only moments of an HCP's time. Similarly, Ireland's first National Maternity Experience Survey^26^ reported that women stated that they did not receive sufficient physical and emotional support immediately postpartum and felt that hospital staff were very busy and not always able to provide help and support.

For many women, positive experiences of care included HCP recognition of their partners as an equal parent as they embarked on parenthood as a couple. Observing their partners treated as an equally responsible caregiver to a newborn sets a tone around expectations; expectations from HCPs around who will bear or share parental duties and expectations of the same within the relationship dynamic. Research with fathers consistently reports a lack of father‐specific support within the maternity care systems. This can range from poor communication with HCPs, to being ignored and side‐lined in maternity settings where they feel treated as visitors.^27^ Our findings align with previous research, which shows that women value partner inclusion as an acknowledgement of their partner's vital supportive role,^28^ and extend beyond this by demonstrating that women perceive the inclusion of their partners as a comforting evolution from out‐dated perceptions that mothers are solely responsible for childcare within the family unit. The positive involvement of fathers by HCPs can also help in the preparation of parents.^29^ However, midwives and key HCPs acknowledged their lack of training and confidence in addressing fathers' needs and the numerous individual, social, cultural and health service factors that can present barriers to engaging fathers in perinatal care.^30^ The recognition of a partner as a parent to the child and support to the woman was echoed in women's views.

With regard to the postpartum period, similar to previous research in which mothers expressed dissatisfaction and disappointment in insufficient postpartum services and information,^31^, ^32^ women described the absence of professional and informational support as surprising and anxiety‐provoking. Women who had access to postpartum supports expressed the reassurance, confidence and peace of mind that these supports gave to them as they transitioned through motherhood. Women who did not have someone to call, somewhere to go or someone who asked and someone who listened postpartum independently suggested that these would have helped them and suggested that such resources would be most beneficial if they were delivered beyond the traditional 6‐week provision of maternal healthcare.

Beyond healthcare provision, women's descriptions of what did or could enable a positive postpartum experience extended to social and organization contexts. Substantial, meaningful paternity leave is a societal, organizational and policy issue, which, together with returning to work and work flexibility, was described by women as enabling shared parental responsibilities, providing opportunities for partners to grow into their own parenting roles and decreasing childcare costs. Paternity leave also has wider implications. One study in Ireland found that the prevalence of paternal postnatal depression was 19.4% in employed fathers who did not receive paternity leave compared with 4.2% in fathers who did receive paternal leave.^33^ All partners in this study were male; however, this finding is equally applicable in all family situations.

Childcare and returning to work were inextricably linked. Childcare costs were a frequently mentioned barrier that excluded women from the workplace. In instances where women wished to return to the workplace, as a matter of financial necessity or for maintaining their individuality, childcare costs could be prohibitive. Lack of flexibility within the workplace was mentioned by women as a source of distress and limited the ability to engage wholly in family life and the maternal role. What worked for women was having access to affordable, near‐to‐home childcare and being able to avail of flexible options in returning to work. However, most often, these options were only available to women with means. Childcare costs to parents in Ireland are among the highest in the OECD.^34^ One Irish study with employed women revealed the complex inequalities experienced by mothers in paid employment,^35^ whilst another study showed the influence that these costs have on women's participation in the workplace.^36^

### 4.1 Limitations

Women's experiences are influenced by culturally based expectations. Women in this study were predominately White‐Irish. Women of diverse ethnic and cultural backgrounds may offer different perspectives on factors that lead to positive maternal well‐being and experiences. Additionally, the data lacked perspectives from single mothers or women in same‐sex relationships as all participants were married or living with male partners.

## 5 CONCLUSION

Although there has been increasing interest in understanding the factors involved in cultivating a positive perinatal experience, research largely remains focused on risk, barriers and illness. The current research explored, from women's perspectives, the factors and resources that do and can support positive health and well‐being through this life experience. The study's findings present multiple avenues suggested by women for improving perinatal experiences that can not only be implemented, primarily at healthcare but also at organizational and policy levels. Organizational or policy changes supporting women's return to work would serve to benefit the financial stability and well‐being of the family unit as a whole, as well as the emotional well‐being of the mother. Women want and benefit from maternal health education that prioritizes informed decision‐making processes and is complemented by the attitudes and support women receive from their HCPs. Women want an active role in their own care and to be active partners in the decisions that affect them and their children. Services and interactions with HCPs that do not facilitate women to make informed, active decisions leave women feeling frustrated and distrustful of both. Interactions with HCPs that validate and encourage women and actively involve their partners left lasting positive impressions on women and set a tone for expectations in motherhood and shared parenting. Additionally, extended and professional postpartum support was a notable and practical consideration, with women suggesting phone lines or drop‐in clinics where women could access HCPs who know how to genuinely approach and listen to women's concerns. When women feel listened to, heard, supported and experience positive care and services in the early postpartum period, they are more likely to embark on motherhood confidently and thrive.

## AUTHOR CONTRIBUTIONS

**Susan Hannon**: Conceptualization; data curation; data analysis; data synthesis; writing – original draft preparation; writing – review and editing. **Elizabeth Newnham**: Conceptualization; data curation; data analysis; data synthesis; writing – original draft preparation; writing – review and editing. **Kathleen Hannon**: Data curation; data analysis; data synthesis; writing – review and editing. **Francesca Wuytack**: Data curation; data analysis; data synthesis; writing – review and editing. **Louise Johnson**: Data synthesis; writing – review and editing. **Ellen McEvoy**: Data synthesis; writing – review and editing. **Déirdre Daly**: Conceptualization; data curation; data analysis; data synthesis; writing – original draft preparation; writing – review and editing. All authors have read and agreed to the published version of the manuscript.

## CONFLICT OF INTEREST

The authors declare no conflict of interest.

## ETHICS STATEMENT

Ethical approval to conduct the study was granted by the Faculty of Health Sciences Research Ethics Committee of Trinity College Dublin on 11 July 2018 (Reference number: 180603).

## Data Availability

The anonymous transcripts that support the findings of this study are available from the corresponding author upon reasonable request that does not contravene the informed consent forms signed by the participants.

## References

[1] WHO . WHO Recommendations on Maternal and Newborn Care for a Positive Postnatal Experience. World Health Organization; 2022. 35467813

[2] WHO . WHO Recommendations on Antenatal Care for a Positive Pregnancy Experience. World Health Organization; 2016. 28079998

[3] WHO . WHO Recommendations: Intrapartum Care for a Positive Childbirth Experience. World Health Organization; 2018. 30070803

[4] Bick D , Duff E , Shakespeare J . Better births—but why not better postnatal care. Midwifery. 2020;80:102574. 31748117 10.1016/j.midw.2019.102574

[5] Buultjens M , Murphy G , Robinson P , Milgrom J , Monfries M . Women's experiences of, and attitudes to, maternity education across the perinatal period in Victoria, Australia: a mixed‐methods approach. Women Birth. 2017;30:406‐414. 28389170 10.1016/j.wombi.2017.03.005

[6] Jenkins MG , Ford JB , Morris JM , Roberts CL . Women's expectations and experiences of maternity care in NSW—what women highlight as most important. Women Birth. 2014;27:214‐219. 24746379 10.1016/j.wombi.2014.03.002

[7] Johansson M , Thies‐Lagergren L , Wells MB . Mothers' experiences in relation to a new Swedish postnatal home‐based model of midwifery care—a cross‐sectional study. Midwifery. 2019;78:140‐149. 31446229 10.1016/j.midw.2019.07.010

[8] Malouf R , Henderson J , Alderdice F . Expectations and experiences of hospital postnatal care in the UK: a systematic review of quantitative and qualitative studies. BMJ Open. 2019;9:e022212. 10.1136/bmjopen-2018-022212 PMC6661900 31320339

[9] Miller YD , Dane AC , Thompson R . A call for better care: the impact of postnatal contact services on women's parenting confidence and experiences of postpartum care in Queensland, Australia. BMC Health Serv Res. 2014;14:635. 25526987 10.1186/s12913-014-0635-9 PMC4301854

[10] Ong SF , Chan WC , Shorey S , Chong YS , Klainin‐Yobas P , He HG . Postnatal experiences and support needs of first‐time mothers in Singapore: a descriptive qualitative study. Midwifery. 2014;30:772‐778. 24161493 10.1016/j.midw.2013.09.004

[11] Sacks E , Langlois ÉV . Postnatal care: increasing coverage, equity, and quality. Lancet Glob Health. 2016;4:e442‐e443. 27185467 10.1016/S2214-109X(16)30092-4

[12] Cronin C , McCarthy G . First‐time mothers—identifying their needs, perceptions and experiences. J Clin Nurs. 2003;12:260‐267. 12603559 10.1046/j.1365-2702.2003.00684.x

[13] McLeish J , Harvey M , Redshaw M , Henderson J , Malouf R , Alderdice F . First‐time mothers' expectations and experiences of postnatal care in England. Qual Health Res. 2020;30:1876‐1887. 32940583 10.1177/1049732320944141 PMC7528544

[14] Finlayson K , Crossland N , Bonet M , Downe S . What matters to women in the postnatal period: a meta‐synthesis of qualitative studies. PLoS One. 2020;15:e0231415. 32320424 10.1371/journal.pone.0231415 PMC7176084

[15] Larkin P , Begley CM , Devane D . Women's experiences of labour and birth: an evolutionary concept analysis. Midwifery. 2009;25:e49‐e59. 17996342 10.1016/j.midw.2007.07.010

[16] Feldman R , Sussman AL , Zigler E . Parental leave and work adaptation at the transition to parenthood: individual, marital, and social correlates. J Appl Dev Psychol. 2004;25:459‐479.

[17] Dow DM . Integrated motherhood: beyond hegemonic ideologies of motherhood. J Marriage Fam. 2016;78:180‐196.

[18] Atzl VM , Grande LA , Davis EP , Narayan AJ . Perinatal promotive and protective factors for women with histories of childhood abuse and neglect. Child Abuse Negl. 2019;91:63‐77. 30831534 10.1016/j.chiabu.2019.02.008 PMC6506345

[19] Vogels‐Broeke M , de Vries R , Nieuwenhuijze M . Dimensions in women's experience of the perinatal period. Midwifery. 2020;83:102602. 31882183 10.1016/j.midw.2019.102602

[20] Shorey S , Ng ED . Application of the salutogenic theory in the perinatal period: a systematic mixed studies review. Int J Nurs Stud. 2020;101:103398. 31678840 10.1016/j.ijnurstu.2019.103398

[21] Daly D , Moran P , Wuytack F , et al. The maternal health‐related issues that matter most to women in Ireland as they transition to motherhood—a qualitative study. Women Birth. 2021;35:10. 10.1016/j.wombi.2021.01.013 33582046

[22] Braun V , Clarke V . Using thematic analysis in psychology. Qual Res Psychol. 2006;3:77‐101.

[23] Baas CI , Erwich JJ , Wiegers TA , de Cock TP , Hutton EK . Women's suggestions for improving midwifery care in The Netherlands. Birth. 2015;42:369‐378. 26467657 10.1111/birt.12185

[24] Tully KP , Stuebe AM , Verbiest SB . The fourth trimester: a critical transition period with unmet maternal health needs. Am J Obstet Gynecol. 2017;217:37‐41. 28390671 10.1016/j.ajog.2017.03.032

[25] Foster W , McKellar L , Fleet JA , Sweet L . Exploring moral distress in Australian midwifery practice. Women Birth. 2022;35:349‐359. 34654667 10.1016/j.wombi.2021.09.006

[26] HSE . Listening, responding and improving. The HSE response to the findings of the National Maternity Experience Survey. October 1, 2020. Accessed June 10, 2022. https://www.hse.ie/eng/services/publications/hse-response-to-the-findings-of-the-national-maternity-experience-survey-2020.pdf2020

[27] Hodgson S , Painter J , Kilby L , Hirst J . The experiences of first‐time fathers in perinatal services: present but invisible. Healthcare. 2021;9:161. 33546202 10.3390/healthcare9020161 PMC7913323

[28] Vedeler C , Nilsen A , Blix E , Downe S , Eri T . What women emphasise as important aspects of care in childbirth—an online survey. BJOG. 2022;129:647‐655. 34532959 10.1111/1471-0528.16926

[29] Walsh TB , Carpenter E , Costanzo MA , Howard L , Reynders R . Present as a partner and a parent: mothers' and fathers' perspectives on father participation in prenatal care. Infant Ment Health J. 2021;42:386‐399. 33955042 10.1002/imhj.21920 PMC9060388

[30] Wynter K , Di Manno L , Watkins V , Rasmussen B , Macdonald JA . Midwives' experiences of father participation in maternity care at a large metropolitan health service in Australia. Midwifery. 2021;101:103046. 34098224 10.1016/j.midw.2021.103046

[31] Schmied V , Cooke M , Gutwein R , Steinlein E , Homer C . Time to listen: strategies to improve hospital‐based postnatal care. Women Birth. 2008;21:99‐105. 18579461 10.1016/j.wombi.2008.04.002

[32] Nan Y , Zhang J , Nisar A , et al. Professional support during the postpartum period: primiparous mothers' views on professional services and their expectations, and barriers to utilizing professional help. BMC Pregnancy Childbirth. 2020;20:402. 32652965 10.1186/s12884-020-03087-4 PMC7353719

[33] Philpott LF , Corcoran P . Paternal postnatal depression in Ireland: prevalence and associated factors. Midwifery. 2018;56:121‐127. 29096280 10.1016/j.midw.2017.10.009

[34] Tuda D . *Childcare in Ireland: Usage, Affordability and Incentives to Work*. ESRI; 2021. ESRI Working Paper 708. https://www.esri.ie/publications/childcare-in-ireland-usage-affordability-and-incentives-to-work

[35] O'Hagan C . Broadening the intersectional path: revealing organizational practices through ‘working mothers’ narratives about time. Gend Work Organ. 2018;25:443‐458.

[36] Kenny O . *Maternal Employment and the Cost of Childcare in Ireland*. ESRI; 2018. ESRI Research Series 73. 10.26504/RS73

